# EEG–ShuffleFormer: A Multi-View Hybrid Network Integrating Time–Frequency and Raw Signal Representations for Few-Channel Motor Imagery EEG Classification

**DOI:** 10.3390/bioengineering13050578

**Published:** 2026-05-19

**Authors:** Kang Fan, Qin Gu, Yaduan Ruan

**Affiliations:** Department of Critical Care Medicine, Nanjing Drum Tower Hospital, Affiliated Hospital of Medical School, Nanjing University, Nanjing 210008, China; kangfan@smail.nju.edu.cn (K.F.); icuguqin@nju.edu.cn (Q.G.)

**Keywords:** few-channel MI EEG, ShuffleNet, Transformer, hybrid network, multi-view feature fusion, continuous wavelet transform, transfer learning

## Abstract

Electroencephalogram (EEG) signals hold significant research value in brain function decoding, disease diagnosis, and brain–computer interfaces (BCIs). Few-channel EEG recording devices feature superior portability, simple operation, and facilitated real-time monitoring implementation. However, few-channel motor imagery (MI) EEG signals inherently suffer from data scarcity and limited spatial discriminative information, which pose critical challenges, including insufficient feature extraction and poor robustness in classification tasks. To address these issues, this paper presents EEG–ShuffleFormer, a hybrid network that integrates two complementary views of EEG signals: time–frequency representations obtained via continuous wavelet transform and the original raw signal representations. A lightweight ShuffleNet backbone extracts local features, followed by a Transformer encoder that models long-range temporal dependencies. Evaluated on the BCI Competition IV Dataset 2b, the proposed method achieves an average classification accuracy of 82.23%, with a substantial improvement on challenging subjects compared to the closest baseline method. Compared with existing methods, the proposed multi-view fusion strategy raises the performance floor while maintaining high accuracy on typical subjects, demonstrating its potential to enhance robustness for different subjects in few-channel scenarios.

## 1. Introduction

Electroencephalogram (EEG) signals are weak electrical signals that reflect human neuronal activities, recorded from the scalp surface using non-invasive devices [1]. As EEG signals can non-invasively reflect the activity state of human brain neurons and their dynamics under external stimuli and specific task conditions without causing trauma to the human body, they have important research value in fields such as neuroscience [2], disease diagnosis (e.g., epilepsy diagnosis, sleep disorders) [3], and brain–computer interfaces (BCIs) [4]. Among them, motor imagery (MI) is one of the core application paradigms in the BCI field, and few-channel MI-EEG acquisition schemes, with the core advantages of strong portability, low cost, and simple deployment, serve as the key carrier to promote BCI technology from laboratory environments to real-world application scenarios, such as home healthcare and consumer-grade wearable devices, thus boasting extremely high practical value and research significance. However, the inherent non-stationarity, low SNR, and inter-individual variability of EEG signals make their robust decoding a long-standing open challenge in the field, and few-channel MI-EEG signals face even more stringent decoding technical bottlenecks due to the limited number of channels.

Traditional feature extraction methods, such as Common Spatial Pattern (CSP) [5], struggle to effectively characterize the dynamic changes in EEG signals and multi-dimensional features, including spatial, temporal, and frequency dimensions, resulting in limited feature representation capabilities. Currently, with the continuous development of deep learning methods, an increasing number of researchers have applied deep learning methods to EEG signal classification research and achieved promising progress. A variety of deep learning-based frameworks have been extensively proposed for MI-EEG classification, including architectures built on convolutional neural networks (CNNs) [6], graph neural networks (GNNs) [7], recurrent neural networks (RNNs) [8], and self-attention (SA) [9].

EEGNet [10] proposes a compact convolutional neural network architecture that takes time-series EEG signals as input. It has demonstrated excellent classification performance in four BCI paradigms, namely P300 visual evoked potentials, error-related negativity, movement-related cortical potentials, and sensorimotor rhythms. To address the defect that conventional methods cannot characterize the topological relationship between electrodes, Xue et al. [11] proposed F-FGCN, a graph neural network model integrating the brain neuronal forward–forward mechanism, which achieved state-of-the-art decoding performance on the PhysioNet MI dataset. Addressing the problem that traditional models rely heavily on manual feature design, Zhang et al. [12] proposed CLRNet, a hybrid model combining CNN, BiLSTM and ResNet, which achieved 89.0% four-class classification accuracy on the BCI Competition IV Dataset 2a. To address the limitation that traditional CNNs are difficult to capture the long-range temporal dependency of signals, Anh Hoang Phuc Nguyen et al. [13] proposed EEG-TCNTransformer, a model integrating a convolutional architecture and multi-head self-attention, which provides a new technical perspective for MI-EEG decoding. Aung et al. [14] proposed an innovative technique for constructing adjacency matrices for EEG channels, named EEG_GLT-Net. This method does not require prior knowledge of inter-channel relationships and can be tailored to suit different subjects and graph neural network architectures. More recently, hybrid architectures combining convolutional and attention-based modules have also been applied to EEG-based emotion recognition tasks, demonstrating the generality of such designs for biomedical signal processing [15]. It is worth noting that the aforementioned methods, particularly GNN-based approaches such as F-FGCN [11], derive much of their advantage from explicitly modeling spatial relationships among electrodes. On high-density arrays with dozens of channels, this topological prior proves highly beneficial. However, in extreme few-channel scenarios such as the 3-channel setup of the BCI IV 2b dataset, the spatial graph reduces to just three nodes, and whether GNN-based modeling retains its advantage in such constrained settings remains an open question.

Most of the aforementioned methods are designed and optimized for multi-channel EEG signal classification tasks, and their feature extraction capability and generalization performance are often subject to certain limitations in few-channel scenarios with a limited electrode count. In few-channel scenarios, the reduction in channel number introduces critical challenges to EEG decoding, including data sparsity and limited spatial discriminative information. How to effectively solve the problem of low classification accuracy caused by data sparsity and limited spatial information in few-channel EEG signals is still one of the core research difficulties in current EEG signal decoding and classification.

Zhang et al. [16] proposed a fast adaptive subband blind source separation technique suitable for short-term, few-channel MI EEG signals, which achieved effective artifact removal. Khademi et al. [17] explored single-channel EEG signal classification through three hybrid models, but their research was limited to analyzing the time–frequency features of a single channel and did not address the problem of information fusion strategies between few-channel signals. Xu et al. [18] proposed a method to fuse the time–frequency maps of three EEG channels in a planar space, but this method has poor scalability and fails to effectively leverage the advantages of deep learning models. Addressing the data scarcity problem of few-channel EEG signals, Ali Al-Saegh et al. [19] proposed a novel EEG dataset augmentation method. Liu et al. [20] proposed converting EEG signals into two-dimensional time–frequency representations and constructing Channel-Dependent Multi-layer EEG time–frequency Representations (CDML-EEG-TFRs), and developed a framework combining an EfficientNet backbone with transfer learning to cope with the data sparsity problem in few-channel scenarios. This CDML-EEG-TFR framework serves as a strong baseline and the methodological starting point for the present study. However, its reliance on a single-view time–frequency input and the weight freezing strategy limit its robustness for subjects whose discriminative features are not well captured by time–frequency representations alone. To address this limitation, the present work investigates whether fusing raw signal information as a complementary view can improve robustness for these challenging subjects.

In addition, existing deep learning methods mainly focus on the single mining of time–frequency features. For example, Xu et al. [18] proposed fusing the time–frequency maps of three channels as the input of a CNN; Zhang et al. [16] extracted features through adaptive subbands; and Liu et al. [20] constructed multi-layer EEG time–frequency representations and combined them with EfficientNet for transfer learning. However, most of the above methods have the limitation of a single feature perspective. They usually directly convert EEG signals into two-dimensional time–frequency maps as the sole input. Although this leverages the advantages of time–frequency features, it often ignores the phase information and amplitude variations in the raw EEG signals. In the context of the inherent sparsity of few-channel data, this single-modality, local-perspective feature extraction method often leads to large fluctuations in the generalization performance of the model when applied to different subjects, making it difficult to achieve ideal results for all individuals.

The classification of few-channel EEG signals faces problems such as limited spatial information and data sparsity. To address these issues, this paper investigates a multi-view feature fusion strategy and constructs a complete classification pipeline for few-channel MI-EEG. Specifically, we design a multi-view feature representation that fuses raw EEG signals with their time–frequency counterparts, preserving discriminative information that time–frequency-only pipelines can lose. This representation is integrated into a hybrid architecture that combines ImageNet-pretrained ShuffleNet [21] for local feature encoding and a Transformer encoder [22] for long-range temporal dependency modeling. The main contributions of this work are as follows:

- Multi-view fusion feature design. We design a feature representation that fuses raw EEG signals with time–frequency features via lightweight 1×1 convolutions. This design retains the amplitude and phase information critical for challenging subjects, without introducing additional model complexity.
- Complete pipeline construction. We construct a fully specified and reproducible processing pipeline—from signal slicing, CWT transformation, and multi-view fusion to hybrid ShuffleNet–Transformer training—providing a practical reference for few-channel MI-EEG classification.
- Systematic ablation and empirical findings. Through three sets of ablation experiments, we quantify the individual contribution of the fusion strategy, transfer learning, and Transformer-based temporal modeling. The results show that the multi-view fusion design is the primary driver of robustness improvement, substantially raising accuracy on the most difficult subject while maintaining performance on all others.

## 2. Materials and Methods

**Figure 1:**
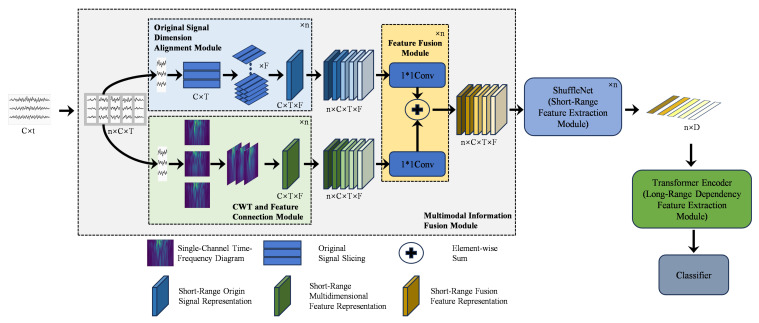
The overall architecture of the proposed EEG–ShuffleFormer model. The framework consists of three main stages: (1) a multi-view Information Fusion module, which separately processes raw EEG signal slices and their corresponding time–frequency representations generated via CWT, and fuses the raw signal features and time–frequency features through 1×1 convolutions to generate an information-rich multi-view feature representation; (2) a ShuffleNet backbone network, which serves as the short-range feature extractor; (3) a Transformer encoder module, which captures long-range temporal dependency features from the feature sequence prior to the final classification.

This paper constructs a classification pipeline for few-channel MI-EEG, termed EEG–ShuffleFormer, which integrates multi-view feature fusion with a hybrid network. The core idea is to retain discriminative information—particularly amplitude and phase cues—that time–frequency transformation alone can lose, especially for challenging subjects. The overall model structure is illustrated in Figure 1.

First, the raw EEG signals are sliced and fed into two distinct modules. The first is the continuous wavelet transform (CWT) and feature concatenation module, which is used to construct short-range time–frequency feature representations, including short-range time–frequency information and channel feature information. The second is the raw signal dimension alignment module, which performs a vertical stacking operation on the raw signals to align the dimensions of the stacked signals with those of the short-range time–frequency features obtained from the previous module, thereby acquiring short-range raw signal features. Next, the obtained short-range time–frequency features and raw signal features are passed through an independent 1×1 convolutional layer before element-wise fusion to obtain the short-range fused feature representation, which integrates multi-view features of the signal such as short-range features, spectral features and raw amplitude features. Subsequently, the sequence of fused short-term feature representations is fed into the ShuffleNet pre-trained on the ImageNet dataset [23] to extract short-range time–frequency features, yielding a two-dimensional feature matrix with long-range temporal dependency features. This matrix is then fed into the Transformer encoder, which leverages its global modeling capability to capture long-range temporal dependency features. Finally, the processed features are input into the classifier for feature classification.

### 2.1. Dataset and Preprocessing

**Figure 2:**
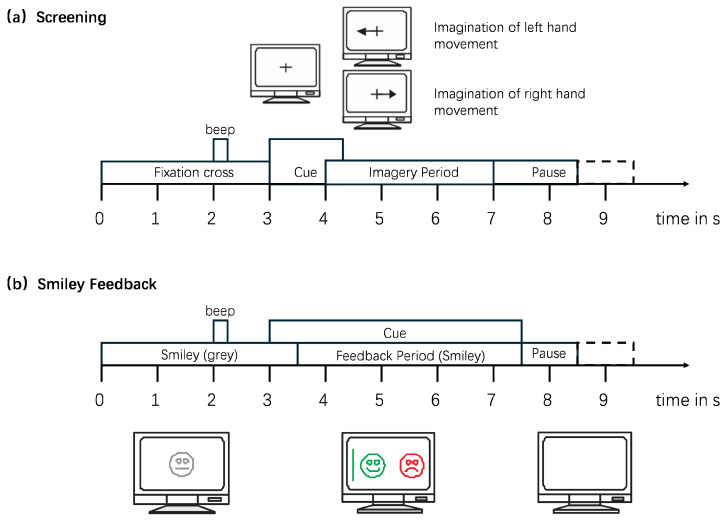
Timing scheme of the paradigm. (**a**) The first two sessions (01T, 02T) contain training data without feedback, and (**b**) the last three sessions (03T, 04E, 05E) with smiley feedback.

The dataset used in this study is the BCI Competition IV Dataset 2b (BCI IV 2b) [24], which is widely used in the field of MI. The BCI IV 2b dataset contains EEG data for two types of MI (left hand and right hand) from 9 subjects. In the experiment, three electrodes (C3, Cz, and C4) were used to record EEG signals. All collected signals were processed with a bandpass filter of 0.5 to 100 Hz and a notch filter of 50 Hz, with a sampling frequency of 250 Hz. The dataset consists of five sessions, among which the first two are EEG data of MI without feedback, the last three are EEG data of MI with feedback, the first three serve as the training set, and the last two as the test set. For the non-feedback experimental data, each subject has 240 trials (120 per MI class). Each experiment lasts 8–9 s, with the MI process occurring from the 3rd second to the 7th second. This is followed by a random interval of 1–2 s as the rest time between different test experiments, and the entire test process is as shown in Figure 2. For the feedback experimental data, each subject includes a total of 480 trials (240 per MI class). Each experiment has a duration of 8–9 s, with the MI process taking place from the 3rd second to the 7.5th second. After that, there is a random interval of 1–2 s as the rest time between different test experiments. The entire experiment process is shown in Figure 2.

To eliminate the interference from environmental noise and physiological artifacts in EEG signals and simultaneously amplify the feature information of key frequencies, this study implemented frequency-band-specific filtering processing on the MI dataset. A 4–40 Hz bandpass filter was applied to the dataset, aiming to retain the μ rhythm (8–13 Hz) and β rhythm (13–30 Hz) related to MI tasks [25], while suppressing high-frequency myoelectric noise (>40 Hz) and ultra-low-frequency drift interference (<4 Hz).

To retain valid data, we segmented the filtered signals, with each segmented fragment serving as an independent time segment corresponding to a classification label. We extracted the 4-second period during which the MI occurred, removing redundant segments unrelated to MI.

### 2.2. Multi-View Information Fusion Module

#### 2.2.1. Channel Configuration and Time Slicing

In this paper, three electrode channels—C3, C4 and Cz—are used. Among them, channels C3 and C4 are located in the left and right primary motor cortex regions of the International 10–20 System, respectively, which directly correspond to the neural activity representation of limb MI (left hand/right hand) [26]. The Cz electrode is located on the central midline and is used to monitor the synergistic activity of bilateral movements. Multiple studies have confirmed that the C3/C4/Cz channel combination has high sensitivity for limb motor classification [27,28].

In this paper, each sample segment is uniformly sliced along the time dimension. A single slice represents short-range information, while the complete time segment composed of multiple slices contains long-range temporal dependency information. In subsequent operations, short-range time–frequency features can be obtained by extracting the local information of a single slice, and the long-range temporal dependency features of the signal can be further obtained by extracting the feature dependencies of multiple time slices. For MI EEG signals, considering the characteristics of short duration and rapid changes in MI, this paper adopts a 0.5-second time segment for feature slicing. Then the signal of the *c*-th channel (c∈C3, C4, Cz) of the *i*-th slice can be expressed as:

(1) $$x_{c}^{\left(i\right)}\in R^{T}$$

where *T* is the number of time points contained in the slice (T=125 for 250 Hz).

#### 2.2.2. CWT and Feature Connection Module

EEG signals exhibit strong non-stationarity and cover multiple frequency bands. To effectively characterize the temporal, frequency and channel features of EEG signals, this paper adopts the CDML-EEG-TFR representation proposed in a previous study [20] to extract the temporal, frequency and channel features of each slice obtained through the above time slicing operation. First, the CWT is used to extract the time–frequency features of the signal, and the signal of each channel in each slice is converted into a time–frequency image $X_{c}^{\left(i\right)}\in R^{T\times F}$ with the horizontal axis representing time and the vertical axis representing frequency:

(2) $$X_{c}^{\left(i\right)}=\mathrm{CWT}\left(x_{c}^{\left(i\right)}\right)$$

**Figure 3:**
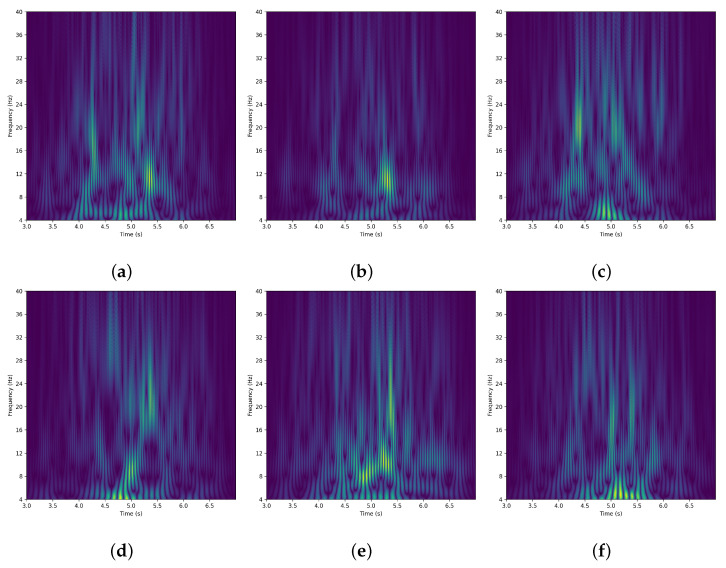
Time–frequency maps of channels (**a**) C3, (**b**) Cz, and (**c**) C4 for left-hand motor imagery, and (**d**) C3, (**e**) Cz, and (**f**) C4 for right-hand motor imagery, during the 3–7 s interval of the MI experiment. The maps are obtained from a single trial of a subject in the BCI IV 2b dataset. The horizontal axis represents time (3–7 s), the vertical axis represents frequency (4–40 Hz), and the color indicates normalized magnitude.

CWT is computed using the complex Morlet wavelet (morl, center frequency $f_{c}\approx 0.8125$ Hz). To match the temporal resolution of the 0.5 s slice (T=125 samples at 250 Hz), the frequency axis is sampled at F=125 linearly spaced points from 4 Hz to 40 Hz, yielding a time–frequency map $X_{c}^{\left(i\right)}\in R^{125\times 125}$. For each target frequency *f*, the corresponding wavelet scale *s* is obtained by

$$s=\frac{f_{c}}{f\cdot \Delta t}=\frac{f_{c}\cdot f_{s}}{f},$$

where Δt=1/250 s and $f_{s}=250$ Hz. The CWT coefficients are computed with $L_{2}$ normalization. Prior to the transformation, a Hamming window of length 125 is applied to each slice to reduce edge artifacts. The magnitude (absolute value) of the complex coefficients is taken as the time–frequency feature representation. The output of the CWT is a two-dimensional matrix, where one dimension represents time and the other represents frequency. On the time–frequency plane, the CWT can reveal the local spectral information of the EEG signal. Figure 3 shows the time–frequency maps of channels C3, Cz and C4 in the BCI IV 2b dataset after filtering and CWT processing during the MI task: the first row corresponds to the left-hand MI state, and the second row corresponds to the right-hand MI state.

Subsequently, a feature concatenation method is adopted to vertically stack the time–frequency image features of different channels to form a channel-correlated multi-layer EEG time–frequency image ${𝓧}^{\left(i\right)}\in R^{C\times T\times F}$, which is similar to an RGB three-channel natural image:

(3) $${𝓧}^{\left(i\right)}=\left[X_{C3}^{\left(i\right)}\left\|X_{\mathrm{Cz}}^{\left(i\right)}\right\|X_{C4}^{\left(i\right)}\right]$$

where ‖ denotes the concatenation operation along the channel dimension. This image has a more comprehensive and rich feature representation, with its three dimensions corresponding to time, frequency and channel, respectively. This representation method can not only well integrate the temporal, frequency and channel features of EEG signals to achieve effective characterization of brain states under the few-channel constraint, but also conform to the input format of CNN, further improving the extraction capability of convolution operations for these three types of feature information. Finally, the CDML-EEG-TFRs formed by multiple slices are combined to construct a multi-dimensional time–frequency representation of EEG signals with temporal dependencies:

(4) $$X=\left({𝓧}^{\left(1\right)},{𝓧}^{\left(2\right)},\ldots ,{𝓧}^{\left(n\right)}\right)$$

This representation contains four dimensions—short-range features, spectral features, spatial features and long-range temporal dependency features—which can characterize the multi-dimensional feature information of few-channel MI EEG signals.

#### 2.2.3. Original Signal Dimension Alignment Module and Feature Fusion Module

For most subjects, the time–frequency representation provides sufficient discriminative information. For a subset of challenging subjects, however, amplitude and phase cues in the raw time-domain signal become more important. To allow the network to draw on either source as needed, this paper introduces a parallel raw time–frequency signal branch and a lightweight fusion mechanism. In contrast to CWT and the Feature Connection Module, we select the raw signal without bandpass filtering. First, the raw signal is normalized. For each slice, the normalized original EEG signal matrix is:

(5) $$X_{\mathrm{raw}}^{\left(i\right)}\in R^{C\times T}$$

where *C* is the number of channels, and *T* is the number of time sampling points within the slice.

The time–frequency branch outputs a tensor of shape C×T×F, while the raw signal is C×T. To bring them into the same shape for fusion, the raw signal is replicated along the frequency dimension:

(6) $${𝓧}_{\mathrm{raw}}^{\left(i\right)}=X_{\mathrm{raw}}^{\left(i\right)}⊗1_{F}^{⊤}$$

where $1_{F}^{⊤}$ is an all-ones row vector of length *F*, and ⊗ denotes the outer product.

The two branches are then each passed through an independent 1×1 convolution and combined via element-wise addition:

(7) $$F^{\left(i\right)}={\mathrm{Conv}}_{1\times 1}\left({𝓧}_{\mathrm{raw}}^{\left(i\right)}\right)+{\mathrm{Conv}}_{1\times 1}\left({𝓧}^{\left(i\right)}\right)$$

The 1×1 convolutions act as per-channel weighting gates. During training, the network learns to emphasize the time–frequency branch when it suffices, and to draw on the raw signal branch when additional amplitude or phase information is beneficial. This adaptive fusion improves robustness for different subjects, particularly for challenging cases, without requiring subject-specific tuning or additional computational overhead.

### 2.3. Hybrid Network Architecture

#### 2.3.1. Short-Range Feature Extraction Module Based on Transfer Learning

The fused feature representations are fed slice by slice into a lightweight CNN backbone for local feature encoding. In few-channel EEG settings, the limited amount of training data makes overfitting a primary concern. A backbone with a smaller capacity is therefore preferable.

ShuffleNet achieves a particularly small model size through pointwise group convolution and channel shuffle operations. The pointwise group convolution reduces the parameter count of 1×1 convolutions, while the channel shuffle enables information exchange across groups without additional parameters. With the smallest width multiplier (0.5×), ShuffleNet contains approximately 0.6M parameters, making it well-suited to data-scarce, few-channel EEG scenarios. For comparison, other lightweight architectures such as ResNet18 and EfficientNet-B0 are also evaluated; the results are discussed in Section 4.2.

To further address the data sparsity problem, this paper adopts the transfer learning strategy proposed in a previous study [20], initializing the backbone with weights pre-trained on ImageNet. This provides a network with low-level feature extraction capabilities learned from natural images, which can be transferred to EEG time–frequency representations and help mitigate overfitting.

Each slice passes through the fused feature generation module to form short-range multi-view fused features. After being processed by ShuffleNet, each short-range fused feature is converted into a *d*-dimensional feature vector:

(8) $$m^{\left(i\right)}=\mathrm{ShuffleNet}\left(F^{\left(i\right)}\right)\in R^{d}$$

The *n* feature blocks finally form a feature matrix with the dimension of d×n:

(9) $$M=\left(m^{\left(1\right)},m^{\left(2\right)},\ldots ,m^{\left(n\right)}\right)\in R^{d\times n}$$

where the row vectors represent the feature dimensions, and the column vectors correspond to the temporal order of the slices.

#### 2.3.2. Long-Range Dependency Feature Extraction Module Based on Transformer

Traditional CNNs are limited by the local receptive field of convolution kernels. In EEG signal processing, they mainly capture short-range features within adjacent time windows, and it is difficult for them to effectively model global correlations, such as cross-second long-range temporal dependency features. In this paper, the aforementioned feature matrix M is taken as the input of the Transformer encoder to model the long-range temporal dependency correlations of the features. The core advantage of Transformer lies in its self-attention mechanism. This mechanism realizes dynamic weight allocation among feature vectors through the interactive computation of query vector (Query, Q), key vector (Key, K) and value vector (Value, V). Its mathematical expression is shown in the following formula:

(10) $$\mathrm{Attention}\left(Q,K,V\right)=\mathrm{softmax}\left(\frac{{\mathrm{QK}}^{T}}{\sqrt{d_{k}}}\right)V$$

where Q, K, and V are obtained by mapping the input sequence through three different linear transformation matrices. This process realizes semantic space transformation of feature vectors and serves as a scaling factor that can effectively prevent the problem of the excessive numerical range of the inner product result of Q and K due to the increase in dimension d, thereby ensuring the gradient stability of attention weights during backpropagation. Through this mechanism, the model can autonomously learn the correlation strength between feature blocks at different time points.

The significant advantages of this design are reflected in two aspects: first, through the cascade of ShuffleNet and Transformer, modeling from local spatial features to global temporal correlations is realized, overcoming the inherent defects of traditional CNN in modeling long-range temporal dependency features; second, the dynamic weight allocation characteristic of the self-attention mechanism enables the model to adaptively focus on feature correlations with physiological significance, thus showing better feature representation ability in EEG tasks.

The model is trained end-to-end using standard cross-entropy loss. Given a mini-batch of *N* samples, let $y_{i}$ denote the true label and ${\hat{y}}_{i}$ be the predicted probability distribution over the two MI classes. The loss function is:

(11) $$L=-\frac{1}{N}\sum_{i=1}^{N}y_{i}\cdot \log{\hat{y}}_{i}$$

## 3. Results

We trained the model on an official training set and tested it on a held-out test set. All model training and testing were completed on a workstation with a fixed hardware configuration. The workstation was equipped with an Intel Xeon Silver 4410Y processor and an NVIDIA GeForce RTX 4090 graphics card (24GB GDDR6X memory). The software environment was built based on an Ubuntu 22.04 LTS operating system, with Python version 3.10.9 and PyTorch framework version 1.13.1. During the training phase, an Adam optimizer was used with an initial learning rate of 0.002; the batch size was set to 32; and the number of training epochs was 50.

We use classification accuracy as the performance metric. Table 1 lists results from several existing studies on the BCI IV 2b dataset alongside the proposed method. Since the code is not open-source, all baseline results are taken directly from their respective original publications; the proposed method is evaluated under the official BCI IV 2b train/test split described in Section 2.1. The proposed pipeline achieves an average accuracy of 82.23%. The per-subject breakdown shows that, compared with the closest baseline (Liu et al. [20], 80.21%), performance is substantially improved on challenging subjects—for Subject 3, its accuracy rises from 54.06% without fusion to 82.30% with the full pipeline—while performance on other subjects remains comparable. It can be seen that the multi-view fusion design raises the performance floor for difficult subjects without significantly degrading the performance of others.

## 4. Discussion

### 4.1. Multi-View Fusion Feature Design

To evaluate the contribution of each view, we compared the full pipeline with two single-view variants: (1) time–frequency-only, where the raw-signal branch was removed, and (2) raw-signal-only, where the signal after dimensional alignment was used as input without the CWT branch. All other components remain unchanged. The results are reported in Table 2.

It should be noted that the two branches operate on different inputs: the time–frequency branch first applies bandpass filtering, whereas the raw-signal branch uses the unfiltered full-band signals. Despite this difference in preprocessing, the performance patterns allow a clear interpretation. Without the raw-signal branch (i.e., time–frequency-only), accuracy drops sharply on several challenging subjects compared to the full model: Subject 3 falls from 82.30% to 54.06%, Subject 2 from 64.05% to 53.70%, and Subject 7 decreases by 7.63%. For subjects that already perform well with time–frequency features alone, the effect of incorporating the raw-signal branch varies: S4, S5, and S8 show only marginal changes, while S9 suffers a notable drop of 7.92% (from 86.58% to 78.66%). Importantly, S9 achieves 86.58% using only time–frequency features—much higher than the 72.34% obtained with raw features alone. This indicates that the bandpass filtering does not discard critical discriminative information; rather, the unfiltered raw signal introduces interference that degrades fusion. The raw-signal branch therefore provides complementary information that is crucial for difficult subjects, but may introduce harmful noise for a small subset of them. Despite this trade-off, the fusion still improves the overall mean accuracy from 77.50% to 82.23% and reduces the between-subject standard deviation from 16.04% to 10.27%, demonstrating better overall robustness.

### 4.2. Short-Range Feature Extraction Module

In this paper, ShuffleNet pre-trained on ImageNet is used to extract short-range feature information of EEG signals. To explore the guiding role of ImageNet prior knowledge in EEG signals, this study compared the performance of the proposed method with and without pre-trained weights, and the results are shown in Table 3.

As can be seen from the data in Table 3, the average accuracy of the model initialized with ImageNet pre-trained weights is 82.23%, which is 5.51% higher than that of the model trained from scratch (76.72%). This proves that prior knowledge learned from the field of natural images can effectively assist in the feature extraction of EEG signals and alleviate problems such as overfitting caused by the data scarcity of few-channel EEG signals.

To identify a suitable backbone for the data-constrained few-channel EEG setting, we compare three lightweight architectures: ResNet18 (11.7M parameters), EfficientNet-B0 (5.3M parameters), and ShuffleNet with a 0.5× width multiplier (0.6M parameters). All backbones are initialized with ImageNet pre-trained weights and trained under identical conditions. The results are shown in Table 4.

Among the three, ShuffleNet 0.5× is designed with channel shuffle and pointwise group convolution, yielding a particularly small capacity. This lightweight design makes it well-suited to few-channel EEG scenarios where the limited amount of training data increases the risk of overfitting. It should be noted that ResNet18 and EfficientNet-B0 do not provide official width-scaled variants comparable to ShuffleNet 0.5×, so a strict capacity-controlled comparison is not feasible. The parameter counts are reported to reflect capacity differences; ShuffleNet’s small capacity is practically advantageous for data-scarce, few-channel EEG settings, without implying architectural superiority.

### 4.3. Long-Range Dependency Feature Extraction Module

To explore the effectiveness of the long-range dependency feature extraction module and the impact of various parameters on performance, this study tested the performance of the model without the Transformer module, and the results are presented in Table 5.

As can be seen from Table 5, the integration of the Transformer has enhanced the feature extraction capability of the network. Compared with the model without Transformer, the average classification accuracy is improved by 2.03%. Due to Transformer’s strong global feature extraction ability, it can extract long-range temporal dependency features hidden in EEG signals, greatly improving the network’s grasp of the global features of EEG signals.

## 5. Conclusions

This paper presents an empirical investigation of a multi-view feature fusion strategy for few-channel MI-EEG classification. Three contributions are made. First, we design a feature representation that fuses raw EEG signals with their time–frequency counterparts via lightweight 1×1 convolutions, preserving amplitude and phase information that time–frequency-only pipelines can discard. Second, we construct a complete and reproducible pipeline—from signal slicing, CWT transformation, and multi-view fusion to hybrid ShuffleNet–Transformer training—providing a practical reference for this data-constrained scenario. Third, through systematic ablation, we quantify the contribution of each component, demonstrating that the fusion design is the primary driver of robustness improvement. On the BCI Competition IV Dataset 2b, the pipeline achieves 82.23% average accuracy, and notably raises the most difficult subject from 54.06% to 82.30%. Compared with prior studies on the same dataset, the proposed pipeline achieves competitive overall accuracy while substantially narrowing the performance gap between challenging and typical subjects—a practical advantage for building equitable BCI systems.

The current pipeline has several limitations that motivate future work. It requires offline preprocessing, which constrains direct application in online BCI settings. In addition, the evaluation is conducted on a single small dataset, and the generalization to larger, more diverse subject populations remains to be validated. Future work will extend this fusion strategy to end-to-end architectures incorporating topology-aware features and validate findings across multiple datasets and larger cohorts.

## Figures and Tables

**Table 1:** Classification accuracies (%) on BCI IV 2b dataset. The best result is marked in bold.

	S1	S2	S3	S4	S5	S6	S7	S8	S9	ACC ± Std
Jiao et al. [29]	76.30	56.00	49.20	**98.20**	91.10	74.80	**88.30**	85.40	84.90	78.20 ± 16.26
Oikonomou et al. [30]	70.63	56.79	58.44	96.25	**91.25**	81.25	73.44	90.63	86.56	78.34 ± 14.40
Al-Saegh et al. [19]	75.31	60.00	60.31	97.19	82.81	**82.50**	74.69	88.13	85.00	78.44 ± 12.33
Moufassih et al. [31]	69.37	60.00	57.19	95.00	80.63	80.00	80.31	91.87	80.63	77.22 ± 12.90
Liu et al. [20]	**77.50**	**67.50**	55.00	98.13	88.75	74.38	86.88	86.88	**86.88**	80.21 ± 13.07
EEG–ShuffleFormer	71.17	64.05	**82.30**	95.89	90.78	80.76	83.57	**92.93**	78.66	**82.23 ± 10.27**

**Table 2:** Performance analysis experimental results of the proposed multi-view fusion method compared to time–frequency features only and raw features only. The best result is marked in bold.

Experiment Content	S1	S2	S3	S4	S5	S6	S7	S8	S9	ACC ± Std (%)
Time–frequency features only	69.37	53.70	54.06	**97.81**	90.31	77.50	75.94	92.19	**86.58**	77.50 ± 16.04
Raw features only	66.81	58.67	78.59	93.68	81.81	71.34	81.20	89.24	72.34	77.08 ±10.98
Proposed method	**71.17**	**64.05**	**82.30**	95.89	**90.78**	**80.76**	**83.57**	**92.93**	78.66	**82.23 ± 10.27**

**Table 3:** Experimental results of the impact of pre-trained weights on performance. The best result is marked in bold.

Experiment Content	S1	S2	S3	S4	S5	S6	S7	S8	S9	Mean ± Std (%)
Without pre-trained weights	64.66	60.27	78.54	**96.36**	74.07	74.55	78.93	89.38	73.72	76.72 ± 11.13
With pre-trained weights	**71.17**	**64.05**	**82.30**	95.89	**90.78**	**80.76**	**83.57**	**92.93**	**78.66**	**82.23 ± 10.27**

**Table 4:** Experimental results of classification performance using different lightweight backbone networks. The best result is marked in bold.

Backbone Network	S1	S2	S3	S4	S5	S6	S7	S8	S9	Mean ± Std (%)
ResNet18	65.72	54.02	73.01	**96.02**	71.47	69.69	76.40	90.46	**78.79**	75.06±12.59
EfficientNetB0	64.65	57.03	69.74	94.10	81.21	65.86	82.60	88.25	78.58	75.78 ±12.17
ShuffleNet	**71.17**	**64.05**	**82.30**	95.89	**90.78**	**80.76**	**83.57**	**92.93**	78.66	**82.23 ± 10.27**

**Table 5:** Experimental results of performance analysis for the long-range feature extraction module. The best result is marked in bold.

Experiment Content	S1	S2	S3	S4	S5	S6	S7	S8	S9	Mean ± Std (%)
Without Transformer	67.13	**70.46**	77.48	**96.40**	82.19	72.44	**84.51**	92.43	**78.72**	80.20 ± 9.81
With Transformer	**71.17**	64.05	**82.30**	95.89	**90.78**	**80.76**	83.57	**92.93**	78.66	**82.23 ± 10.27**

## Data Availability

The data presented in this study are available in BCI Competition IV at https://www.bbci.de/competition/iv/ (accessed on 4 January 2024), reference number [24].

## Abbreviations

The following abbreviations are used in this manuscript:

EEG	Electroencephalogram
CDML-EEG-TFRs	Channel-dependent multi-layer EEG time–frequency representations
BCI	Brain–computer interface
MI	Motor imagery
CNNs	Convolutional neural networks
GNNs	Graph convolutional networks
RNNs	Recurrent neural networks
LSTMs	Long short-term memory networks
CWT	Continuous wavelet transform
